# Virtual Care Remuneration Policy and Postdischarge Follow-Up Trends

**DOI:** 10.1001/jamanetworkopen.2026.20021

**Published:** 2026-06-24

**Authors:** David D’Arienzo, Sanjay Mahant, Peter C. Austin, Yulika Yoshida-Montezuma, Astrid Guttmann

**Affiliations:** 1 Department of Pediatrics, McGill University, Faculty of Medicine and Health Sciences, Montreal, Quebec, Canada; 2 Institute of Health Policy, Management and Evaluation, Dalla Lana School of Public Health, University of Toronto, Toronto, Ontario, Canada; 3 Edwin S. H. Leong Centre for Healthy Children, University of Toronto, Toronto, Ontario, Canada; 4 Department of Paediatrics, University of Toronto Temerty Faculty of Medicine, Toronto, Ontario, Canada; 5 Child Health Evaluative Sciences, The Hospital for Sick Children Research Institute, Toronto, Ontario, Canada; 6 ICES, Toronto, Ontario, Canada; 7 Sunnybrook Research Institute, Toronto, Ontario, Canada

## Abstract

**Importance:**

Timely follow-up after pediatric hospitalization is critical to support safe discharge, yet many children face barriers to in-person visits. Virtual care may facilitate follow-up; however, its association with follow-up trends remains unknown.

**Objective:**

To examine whether a system-wide virtual care remuneration policy, characterized by virtual care physician billing codes, was associated with changes in 7-day postdischarge follow-up rates overall and by hospitalization type and equity factors.

**Design, Setting, and Participants:**

This population-based, repeated cross-sectional study used administrative data on all children aged 18 years or younger with provincial health insurance in Ontario, Canada, who were discharged home after medical or surgical hospitalization between March 1, 2011, and June 30, 2024.

**Exposures:**

The primary exposure was the implementation of the virtual care remuneration policy (instituted December 1, 2022); effects were stratified by hospitalization type, rurality, and socioeconomic status.

**Main Outcomes and Measures:**

The primary outcome was the monthly rate of physician follow-up within 7 days of hospital discharge. The absolute difference in observed vs estimated follow-up visits per 1000 discharges was estimated using an interrupted time series analysis with autoregressive integrated moving average model.

**Results:**

The study included 643 156 hospital discharges across 469 066 children (mean [SD] age, 6.3 [5.7] years; 55.1% male). Before the virtual care policy, 42.0% of children received follow-up within 7 days of discharge vs 40.9% thereafter (follow-up provided virtually in 0.2% of postdischarge visits before the virtual care policy vs 11.0% after implementation). After policy implementation, follow-up remained lowest among rural (29.8%) and the most materially deprived populations (39.0%). Accounting for seasonality and autocorrelation, no significant change in slope (0.36; 95% CI, −0.15 to 0.87) or level (−2.70; 95% CI, −7.09 to 1.69) of follow-up rates were observed after policy implementation. Observed follow-up rates did not differ from those estimated (1 fewer visit per 1000 discharges). No clinically meaningful change in slope or level of follow-up rates were observed by hospitalization type, rurality, or socioeconomic status.

**Conclusions and Relevance:**

In this cross-sectional study of Ontario children discharged from the hospital, a virtual care remuneration policy was not associated with an increase in timely follow-up after pediatric hospitalization, nor was it associated with narrowing of geographic and socioeconomic disparities. Virtual care billing policies alone may be insufficient to enhance postdischarge follow-up and may reinforce existing inequities.

## Key Points

**Question:**

Was the implementation of a system-wide virtual care remuneration policy associated with changes in timely postdischarge follow-up among pediatric patients in Ontario, Canada?

**Findings:**

In this cross-sectional study of 643 156 hospital discharges across 469 066 children, there was no meaningful change in follow-up rates after virtual care remuneration policy implementation overall or by hospitalization type, patient rurality, and socioeconomic status. Rural populations and the most materially deprived populations experienced the lowest rates of virtual follow-up after policy development.

**Meaning:**

This study suggests that a virtual care remuneration policy, characterized by virtual care billing codes, may be insufficient to enhance follow-up in the hospital-to-home transition and may reinforce existing inequities.

## Teaser

This cross-sectional study examines whether a system-wide virtual care remuneration policy was associated with changes in 7-day postdischarge follow-up rates overall and by hospitalization type, patient rurality, and socioeconomic status.

## Introduction

The transition from hospital to home is a vulnerable period for children, with high frequencies of preventable adverse events, including rehospitalizations and emergency department visits.^1,2,3,4^ Timely follow-up after discharge is a key component to safe discharge planning and may help address patient safety risks during the transition to home.^5,6^ Timely follow-up enables clinicians to reassess symptoms, ensure medication adherence, evaluate treatment response, provide patient education, and coordinate specialty services.^7^

National health care organizations recommend timely postdischarge follow-up for most hospitalized patients, with many guidelines recommending follow-up within 7 days of discharge.^8,9,10,11^ Evidence also suggests that earlier follow-up, within 7 days, is more strongly associated with improved health outcomes compared with later follow-up.^12,13^ Despite its importance, obtaining in-person, timely postdischarge follow-up remains challenging, with 28% to 62% of children (across multiple jurisdictions) missing scheduled postdischarge visits.^14^

Virtual care has emerged as a promising tool to facilitate postdischarge follow-up, as it may address barriers to obtaining in-person care, such as transportation difficulties, scheduling limitations, and associated patient costs.^15,16,17,18,19,20^ Pediatricians and families believe that virtual postdischarge follow-up may help optimize the hospital-to-home transition, and 85% of families are interested in using virtual care during the transition.^21,22^ Health care organizations across North America and internationally now recommend virtual care to facilitate postdischarge visits.^6,23,24^ However, virtual care may inadvertently exacerbate inequities in postdischarge follow-up.^25,26,27,28,29,30^ Studies in Ontario, Canada, have shown that individuals living in areas of higher material deprivation report lower comfort with and use of virtual care and that uptake of virtual care is greater among urban physicians and patients compared with their rural counterparts.^25,26,29^

Despite growing enthusiasm for virtual care, it remains unknown whether its availability, enabled through new physician remuneration policies, improves access to timely postdischarge follow-up. In addition, although virtual care initially improved access to care for rural and underserved communities, there are now concerns regarding the equity of virtual care delivery.^25,26,29,31,32^

In this study, we describe trends in timely postdischarge follow-up rates among hospitalized children from 2011 to 2024 in Ontario, Canada. The implementation of a virtual care remuneration policy, characterized by physician billing codes, aimed to improve access to care and encourage the adoption of virtual care. This provided a unique opportunity to examine whether the virtual care remuneration policy was associated with changes in timely postdischarge follow-up rates among hospitalized children and if the changes varied across hospitalization type and equity factors.

## Methods

### Study Design and Population

In this population-based, repeated cross-sectional study using an interrupted time series analysis, we identified all children aged 18 years or younger with provincial health insurance in Ontario, Canada, who were discharged home after an unplanned hospitalization between March 1, 2011, and June 30, 2024. We excluded hospitalizations with case-mix group indicating pregnancy or newborn birth, psychiatric hospitalizations, and readmissions that occurred within 30 days of an index hospitalization discharge. We used linked data from several population-level administrative databases held at ICES, an independent nonprofit research institute. The datasets are linked at ICES using unique encoded identifiers. Its legal status under Ontario’s health information privacy law allows it to collect and analyze health care and demographic data, without consent, for health system evaluation and improvement. The use of these encoded data is authorized under section 45 of Ontario’s Personal Health Information Protection Act, which does not require review by a Research Ethics Board. The study followed the Strengthening the Reporting of Observational Studies in Epidemiology (STROBE) reporting guideline.^33^

### Setting

Ontario operates under a single-payer universal health care system and serves a large, diverse population across densely populated urban centers and remote rural regions. The study period was divided into 3 eras: (1) pre–virtual care era (March 1, 2011, to February 29, 2020), where virtual care billing codes were highly restrictive and virtual visits accounted for less than 2% of all ambulatory care encounters^34,35^; (2) temporary virtual care era (June 1, 2020, to November 30, 2022), characterized by the introduction of temporary virtual care billing codes that had minimal restrictions and equal remuneration as in-person visits^35,36^; and (3) current virtual care era (December 1, 2022, to June 30, 2024), characterized by the development of the current and permanent virtual care billing codes that reimburse 85% of the corresponding in-person rate for telephone visits and 100% for video visits and restricted eligibility to enrolled or rostered patients (for primary care) or to those who had received in-person care from the physician within the preceding 24 months.^37^

### Data Sources

We used the Canadian Institute of Health Information’s Discharge Abstract Database to capture hospitalizations and the National Ambulatory Care Reporting System to capture emergency department records. The Registered Persons Database provided demographic data, including rurality, of all Ontario residents with provincial health insurance. The Ontario Health Insurance Plan database, Community Health Centre, and Client Agency Program Enrollment captured outpatient physician visits, patient-physician enrollment, and modality of visit. The Ontario Marginalization Index derived from census data captured neighborhood material resource quintile at the level of the dissemination area (400-700 people).

### Exposure

The primary exposure was the implementation of the system-wide virtual care remuneration policy (instituted December 1, 2022) for all publicly funded physicians in Ontario, Canada, comparing the current virtual care era (December 1, 2022, to June 1, 2024) with the pre–virtual care era (March 1, 2011, to February 29, 2020).

Data during the temporary virtual care era (June 1, 2020, to November 30, 2022) were described but excluded from the analysis, as this period coincided with the onset of the COVID-19 pandemic and numerous system-level changes (ie, decrease in access to primary care, changes in health care–seeking behaviors, and changes in type and number of hospitalizations), which could have had an independent association with postdischarge follow-up patterns.^35,38,39,40^ For this descriptive analysis, a 3-month washout period (March 1 to May 31, 2020) was applied to allow for the development of virtual care infrastructure.^38,41^

### Outcomes

The primary outcome was the monthly rate of physician follow-up visits within 7 days of hospital discharge, calculated as the number of follow-up visits per month divided by the total number of monthly hospital discharges. Only 1 follow-up visit per patient (earliest visit) with any physician was included. The clinician type and modality of follow-up were also described.

### Statistical Analysis

Descriptive statistics were used to describe the population in each era. Baseline characteristics between the pre–virtual care era and each virtual care era were compared using standardized mean differences (differences <10% were considered unimportant).^42^

We used an interrupted time series analysis to examine for an association between the virtual care remuneration policy and the monthly 7-day postdischarge follow-up rate. Autoregressive integrated moving average (ARIMA) models, without inclusion of additional covariates, were used to account for autocorrelation and seasonality.^43^ Time series forecasting based on ARIMA models have been used to evaluate large-scale health policy interventions.^43,44,45^ The ARIMA models were fitted to the pre–virtual care era data and used to estimate expected postdischarge follow-up rates during the current virtual care era, had there been no implementation of the virtual care policy. Stationarity of the time series was assessed using the augmented Dickey-Fuller test, and appropriate orders of differencing were applied.^43^ Autoregression was incorporated to minimize the Akaike information criterion on the autocorrelation plots, and lagged error terms were incorporated to minimize the Akaike information criterion on the partial autocorrelation plots.^43^ The presence of autocorrelation was then assessed using the Ljung-Box test.^43^

Observed monthly follow-up rates that were outside the 95% CIs of the estimated follow-up rates were interpreted as statistically significant deviations, therefore associated with the virtual care remuneration policy. The absolute differences between the observed and estimated follow-up visits per 1000 hospital discharges were calculated. Differences in slope (rate of change over time) and level change (immediate shift or stepwise change in outcome at the time of the intervention) between the current and pre–virtual care eras were presented as point estimates and 95% CIs.

Analyses were conducted for the overall study population and stratified by (1) hospitalization type, defined by the Canadian Institute for Health Information case-mix group (medical and surgical); (2) rurality, defined by the Rurality Index for Ontario score (≥40 indicates a rural setting)^46,47^; and (3) material resource score, a marker of neighborhood socioeconomic status, which combines census data on income, unemployment, household condition, education, and single-parent families.^48,49,50^ Stratified analyses were conducted as virtual care uptake is hypothesized to vary across these groups. Sensitivity analyses were conducted (1) using monthly age- and sex-standardized rates of follow-up instead of the crude rates of follow-up that are already reported and (2) excluding those readmitted within 7 days of hospital discharge. Unadjusted rates were used in the primary analysis to calculate the absolute difference in observed vs estimated follow-up rates. All statistical analysis was performed using SAS Enterprise Guide, version 7.1 (SAS Institute Inc). All *P* values were from 2-sided tests and results were deemed statistically significant at *P* < .05.

## Results

Over the study period from March 1, 2011, to June 30, 2024, there were 643 156 hospital discharges across 469 066 children (mean [SD] age, 6.3 [5.7] years; 55.1% male and 44.9% female). Most discharges were for medical conditions (n = 464 550 [72.2%]), followed by surgical conditions (n = 178 606 [27.8%]). Baseline characteristics of age, sex, hospitalization type, rurality, material resource quintile, primary care attachment, and hospitalization length of stay were similar across the pre–virtual care and current virtual care eras, with standardized differences less than 0.1 (Table).

**Table.** Annualized Baseline Characteristics of Ontario Children Discharged From Hospital by Virtual Care Era^a^

Characteristic	Pre–virtual care era (March 1, 2011, to February 29, 2020)	Temporary virtual care era (June 1, 2020, to November 30, 2022)	SMD between pre–virtual care and temporary virtual care eras^b^	Current virtual care era (December 1, 2022, to June 30, 2024)	SMD between pre–virtual care and current virtual care eras^b^
No. per year	50 429	40 896	NA	50 568	NA
Age, median (IQR), y	5.0 (1-11)	5.0 (1-12)	0.032	5.0 (1-11)	0.070
Sex, No. (%)					
Female	22 507 (44.6)	18 625 (45.5)	0.018	22 759 (45.0)	0.007
Male	27 922 (55.4)	22 271 (54.5)	0.018	27 819 (55.0)	0.007
Hospitalization type, No. (%)					
Medical	36 097 (71.6)	39 621 (72.4)	0.019	38 193 (72.3)	0.009
Surgical	14 332 (28.4)	11 262 (27.6)	0.019	12 310 (27.7)	0.009
Rurality, No. (%)					
Rural	3435 (6.8)	2396 (6.6)	0.010	3234 (6.4)	0.017
Urban	46 993 (93.2)	38 213 (93.4)	0.010	47 440 (93.6)	0.017
Material resource quintile, No. (%)^c^					
1 (Least deprived)	9315 (18.5)	7281 (17.8)	0.017	8926 (17.6)	0.022
2	9753 (19.3)	8749 (21.4)	0.051	10 741 (21.2)	0.047
3	9350 (18.5)	7989 (19.5)	0.025	10 108 (20.0)	0.037
4	9403 (18.7)	7262 (17.8)	0.023	8996 (17.8)	0.033
5 (Most deprived)	12 608 (25.0)	9616 (23.5)	0.035	11 824 (23.4)	0.038
Primary care attachment, No. (%)					
Not rostered	4640 (9.2)	4972 (11.3)	0.095	6241 (10.8)	0.098
Rostered	45 789 (90.8)	35 924 (87.8)	0.068	44 337 (89.2)	0.078
Length of stay, median (IQR), d	2 (1-3)	2 (1-3)	0.036	2 (1-3)	0.011

Abbreviations: NA, not applicable; SMD, standardized mean difference.

^a^ The pre–virtual care era reflects a period of minimal virtual care use and highly restrictive virtual care billing codes. The current virtual care era reflects the onset of the system-level virtual care remuneration policy.

^b^ SMD greater than 0.1 may indicate meaningful differences between groups.

^c^ Material resource, a marker of neighborhood socioeconomic status, combines income, unemployment rates, household condition, education level, and single-parent family rates. Material resource quintile and hospitalization type had less than 0.02% missing values. All other variables had no missing data.

### Follow-Up Within 7 Days of Discharge

Follow-up within 7 days of discharge occurred for 42.0% of hospitalized patients in the pre–virtual care era and 40.9% during the current virtual care era (eTable 1 in Supplement 1). The 7-day postdischarge follow-up rate was highest among children hospitalized for medical conditions. During the current virtual care era, children residing in rural areas had lower 7-day follow-up rates compared with those residing in urban areas (29.8% vs 41.7%), while 7-day follow-up rates were lowest in the most deprived material resource quintile (39.0% vs 40.7%-42.2%) (eTable 1 in Supplement 1). The 7-day hospital readmission rate was low overall (3.4%), 3.3% in the pre–virtual care era and 3.4% in the post–virtual care era.

### Follow-Up by Clinician Type and Modality

The distribution of clinician type conducting postdischarge follow-up was similar across all study periods (eTable 2 in Supplement 1). In the current virtual care era, pediatricians provided most of the follow-ups for medical hospitalization (58.9%), followed by general practitioners (26.2%).

The proportion of virtual 7-day postdischarge follow-up increased after virtual care remuneration policy implementation. Prior to its implementation, 0.2% of postdischarge visits were virtual compared with 29.7% during the temporary virtual care era and 11.0% in the current era (eTable 3 in Supplement 1). The increase in virtual uptake in the pre–virtual care era vs the current virtual care era was most notable among children hospitalized for medical conditions (0.2% to 10.3%) and surgical conditions (0.2% to 13.9%) and those living in urban areas (0.1% to 10.9%). Among rural populations, the proportion of virtual follow-up increased from 0.0% to 10.7%. In the pre–virtual care era, virtual care comprised 0.1% of postdischarge follow-up visits in the highest socioeconomic status quintile and 0.2% in the lowest quintile. In the current virtual care era, virtual follow-up accounted for a higher proportion of follow-up visits in the highest socioeconomic status quintile compared with the lowest (11.8% vs 9.5%).

### Change in Follow-Up Rates With Onset of a System-Level Virtual Care Remuneration Policy

**Figure 1.**
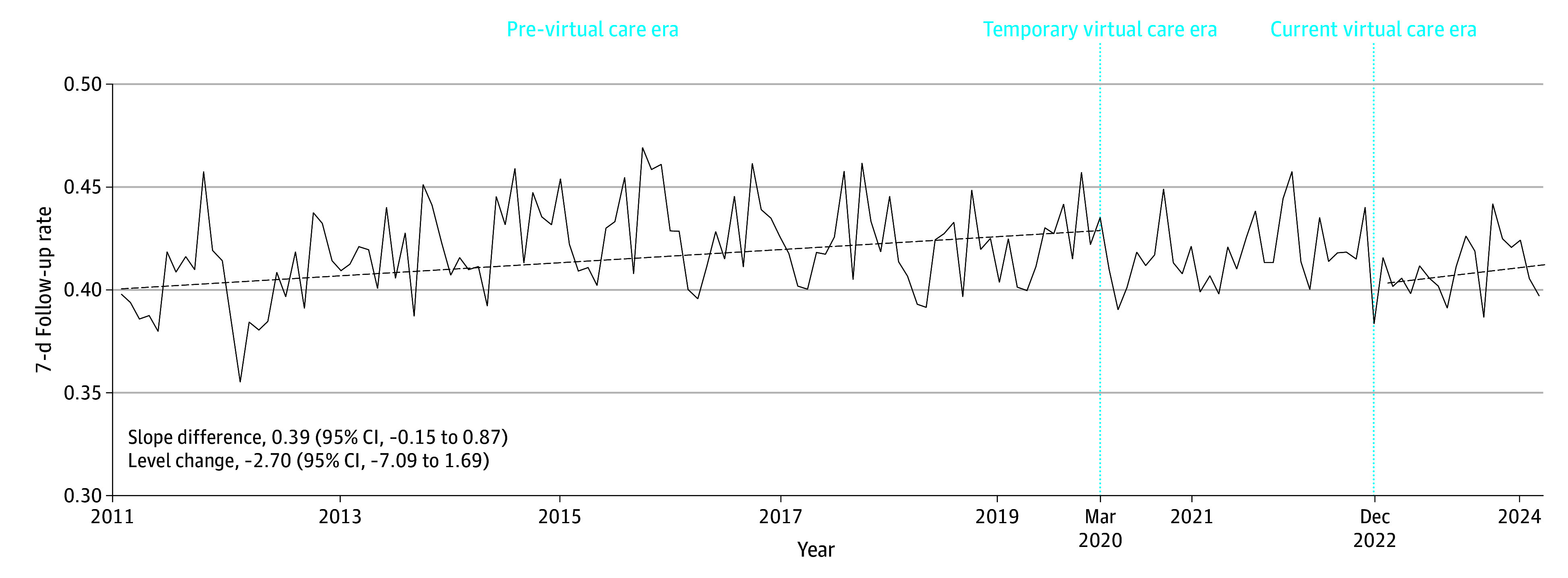
Line Graph Showing Monthly Rate of 7-Day Postdischarge Follow-Up for All Children Hospitalized in Ontario, Canada, Between March 1, 2011, and June 30, 2024 The dotted vertical lines indicate the onset of the temporary virtual care era (March 1, 2020) and the current virtual care era (December 1, 2022) in Ontario. The current virtual care era represents the onset of the system-level virtual care remuneration policy. The monthly 7-day postdischarge follow-up rate was calculated by the total number of patients who received follow-up within 7 days of hospital discharge per total number of hospital discharges per month.

In the pre–virtual care era, the monthly 7-day postdischarge follow-up rate was increasing slightly (0.28; 95% CI, 0.02-0.43) (Figure 1; eTable 4 in Supplement 1). After the implementation of the virtual care remuneration policy, the follow-up rate was neutral (0.58; 95% CI, −0.13 to 1.29). After accounting for seasonality and autocorrelation, there was no significant change in the slope (monthly rate of change) (0.36; 95% CI, −0.15 to 0.87; *P* = .16) or level (−2.70; 95% CI, −7.09 to 1.69; *P* = .23) of postdischarge follow-up rates from the pre–virtual care era to current virtual care era.

**Figure 2.**
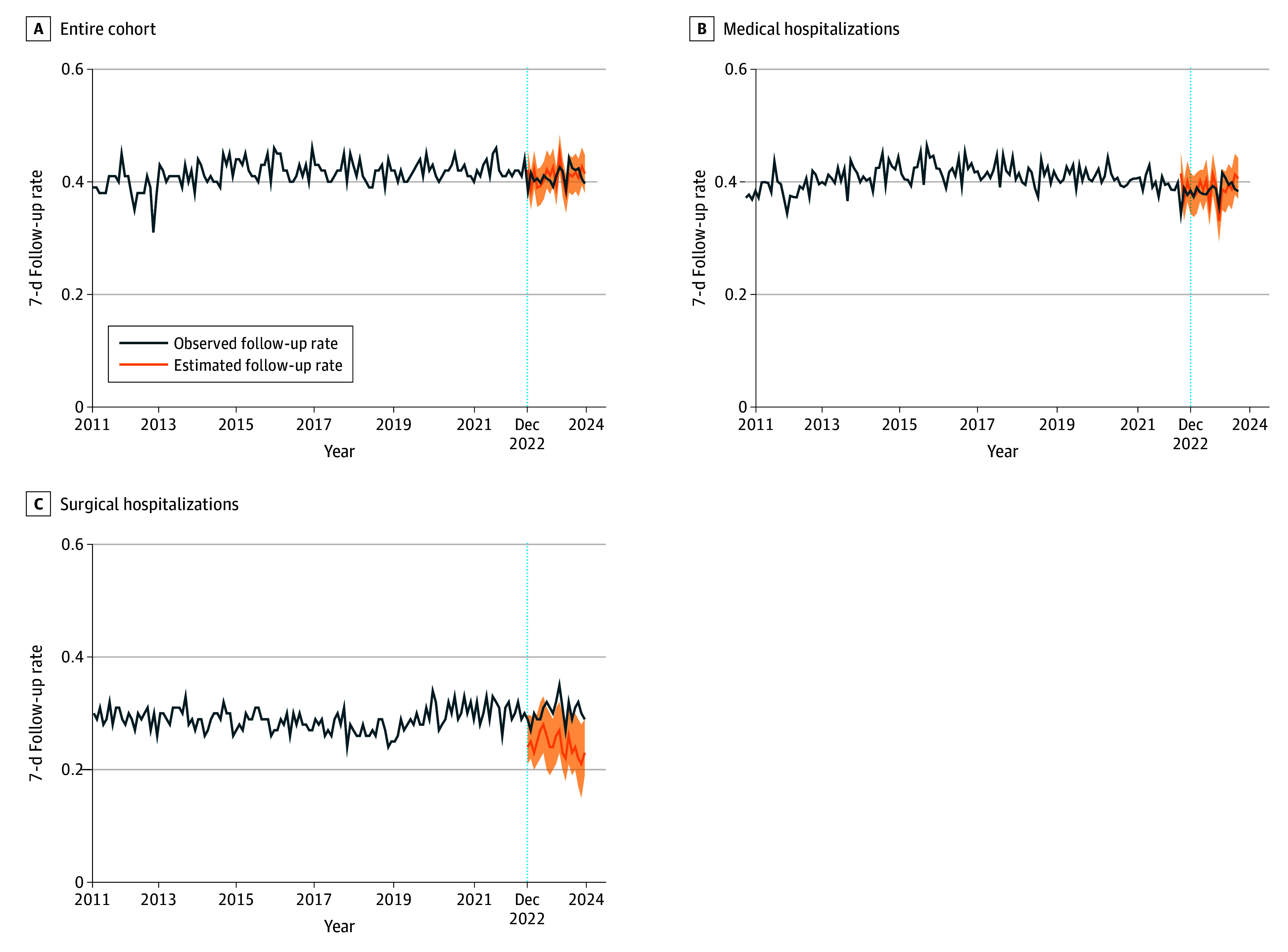
Line Graphs Showing Observed vs Estimated 7-Day Postdischarge Follow-Up Rates From 2011 to 2024 and After the Introduction of the System-Level Virtual Care Remuneration Policy, Overall and by Hospitalization Type The dotted vertical lines indicate the onset of the system-level virtual care remuneration policy and current virtual care era (December 1, 2022) in Ontario. Shaded areas indicate the 95% CIs of the estimated postdischarge follow-up rate.

**Figure 3.**
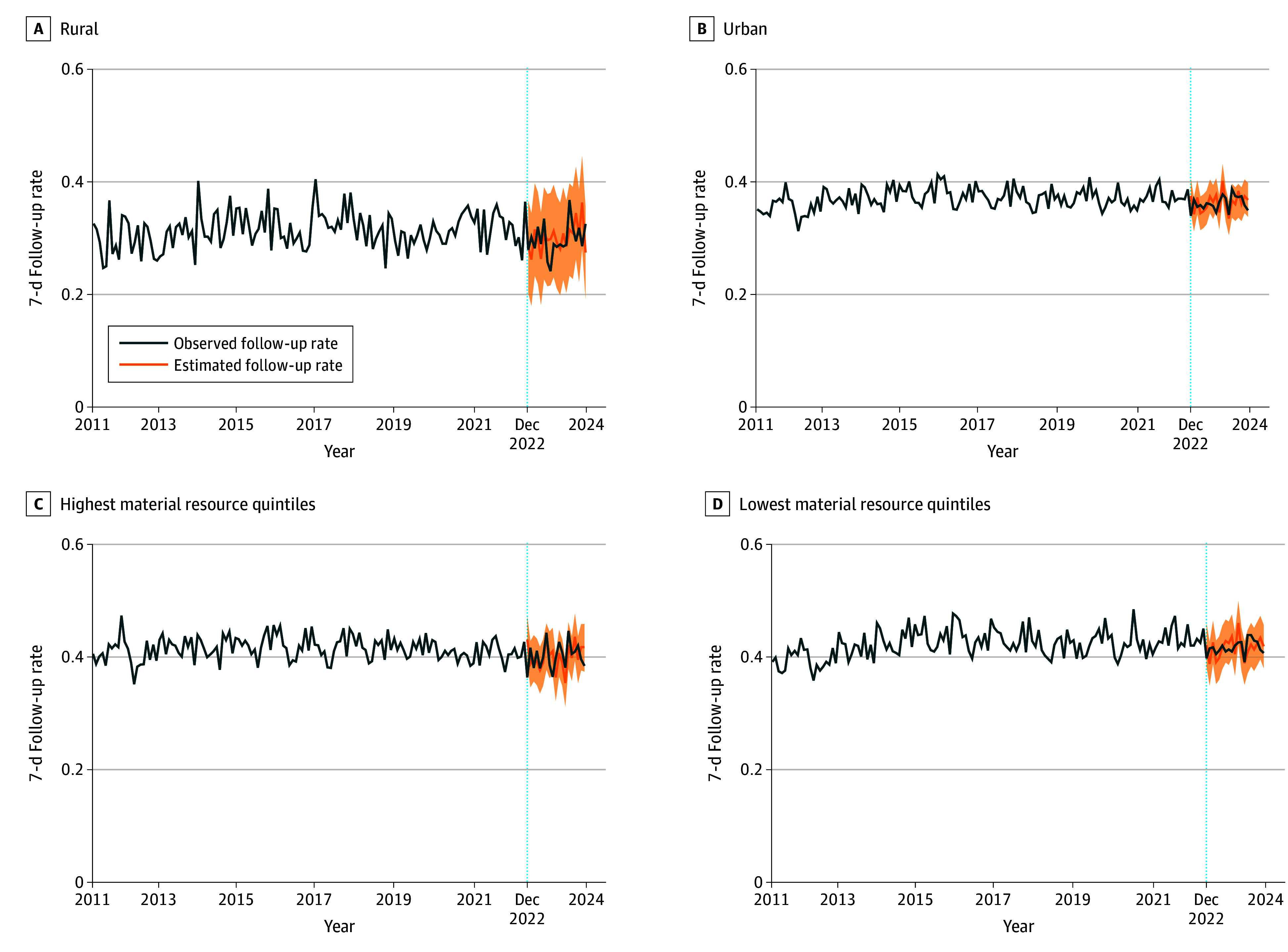
Line Graphs Showing Observed vs Estimated 7-Day Postdischarge Follow-Up Rates From 2011 to 2024 and After the Introduction of the System-Level Virtual Care Remuneration Policy, by Equity Factors The dotted vertical lines indicate the onset of the system-level virtual care remuneration policy and current virtual care era (December 1, 2022) in Ontario. Shaded areas indicate the 95% CIs of the estimated postdischarge follow-up rate.

**Figure 4.**
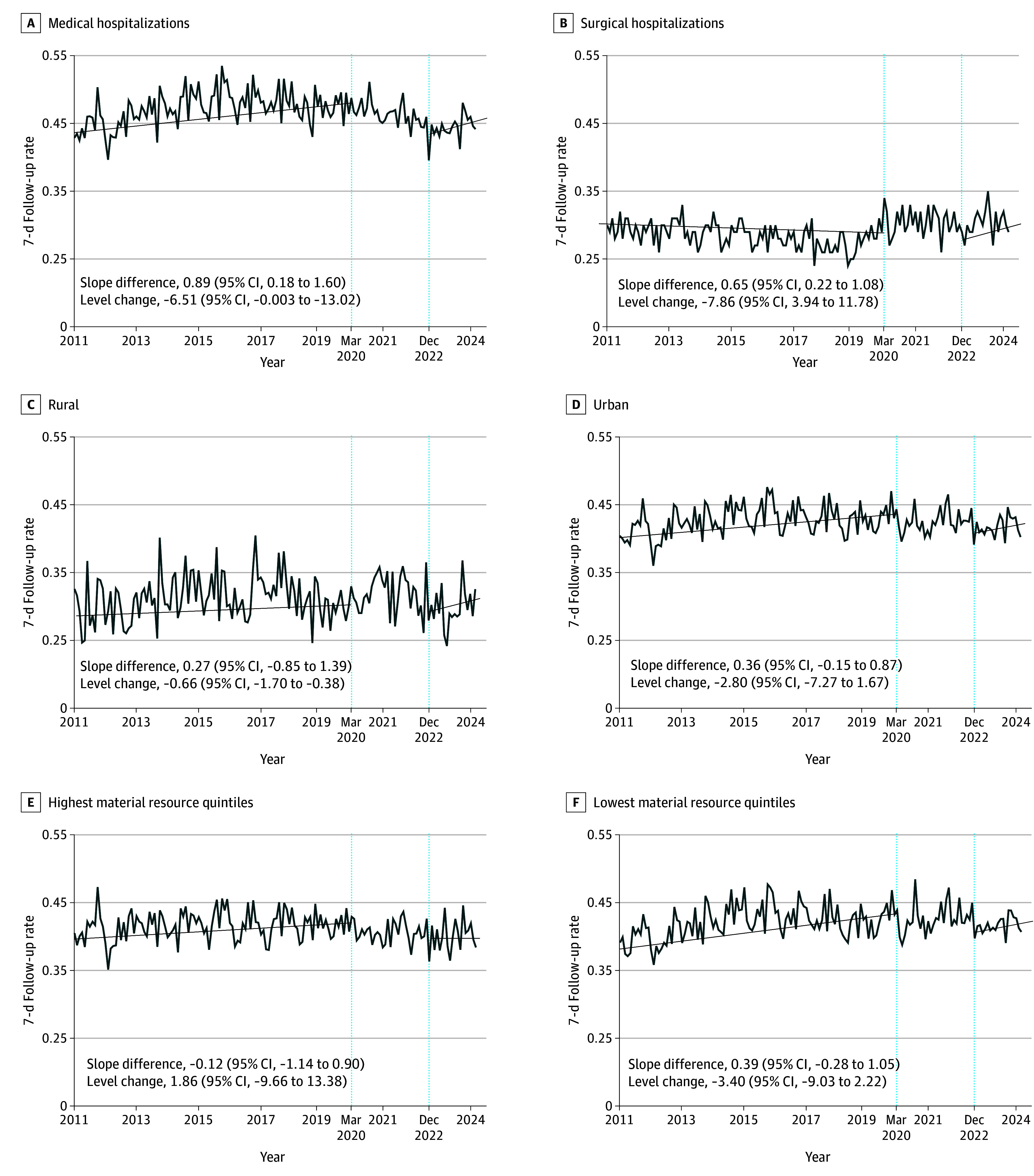
Line Graphs Showing Monthly Rate of 7-Day Postdischarge Follow-Up for Children Hospitalized in Ontario, Canada, Across Virtual Care Eras, Between March 1, 2011, and June 1, 2024, Stratified by Hospitalization Type, Rurality, and Material Resource Score The dotted vertical lines indicate the onset of the temporary virtual care era (March 1, 2020) and current virtual care era (December 1, 2022) in Ontario. The current virtual care era represents the onset of the system-level virtual care remuneration policy. The monthly 7-day postdischarge follow-up rate was calculated by the total number of patients who received follow-up within 7 days of hospital discharge per total number of hospital discharges per month.

Using pre–virtual care era data, the mean monthly estimated follow-up rate in the current virtual care era was 0.41 (range, 0.35-0.48) (Figure 2). The mean monthly observed follow-up rate was 0.41 (range, 0.38-0.44). This corresponds to a negligible difference of 1 fewer follow-up visit per 1000 hospital discharges during the current virtual care era compared with that estimated based on historical trends if no virtual care remuneration policy had been instituted (eTable 5 in Supplement 1). Similar clinically negligible differences were noted in the difference between observed vs estimated follow-up rates across hospitalization types, rurality, and material resource groups (Figure 2 and Figure 3; eTable 5 in Supplement 1). Furthermore, when stratified by hospitalization type, rurality, and material resource score, there were also no clinically meaningful changes in slope or level of follow-up rate across the pre–virtual care era and the current virtual care era (Figure 4; eTable 4 in Supplement 1).

Sensitivity analyses (1) using age- and sex-standardized monthly 7-day postdischarge follow-up rates and (2) excluding patients who were readmitted within 7 days of discharge demonstrated no clinically meaningful changes in either the level or slope of follow-up rates between the pre–virtual care and current virtual care eras overall and stratified by hospitalization type, rurality, and socioeconomic status (eTable 6 and eTable 7 in Supplement 1).

## Discussion

In this population-based, repeated cross-sectional study using an interrupted time series design, we analyzed 643 156 pediatric hospital discharges across Ontario, Canada, from 2011 to 2024. Implementation of a system-level virtual care remuneration policy and virtual care billing codes was not associated with a clinically meaningful change in the rate of 7-day postdischarge follow-up visits overall or by hospitalization type, rurality, or socioeconomic status. In addition, postdischarge follow-up remained lowest among rural populations and those living in the most materially deprived areas after policy implementation. These findings suggest that virtual care availability may not fulfill its anticipated benefits in facilitating postdischarge follow-up or reducing inequities.

Fragmented inpatient-outpatient communication, financial constraints, and long wait times are common barriers to obtaining timely postdischarge follow-up.^15,16,51,52,53,54^ Although virtual care may improve convenience, limited primary care capacity, poor communication systems across care settings, lack of digital infrastructure, and inequities in digital literacy may have been associated with unchanged timely postdischarge follow-up rates. In addition, follow-up by general practitioners decreased by 5% or more across all hospitalization types in the current vs pre–virtual care era, potentially reflecting the increasing number of people in Ontario without a family physician after the COVID-19 pandemic.^55,56^ Although the virtual care policy was associated with an increase in virtual postdischarge visits, its availability and modest uptake (11.0% of follow-up visits) was not associated with an increase in patients receiving timely follow-up. Virtual care billing policies alone may not be sufficient to drive meaningful improvements in care access during the transitional care processes. Financial incentives have also not been associated with substantial improvements in postdischarge follow-up rates.^57,58^ Improving the transitional care process may require a comprehensive, multifaceted approach that addresses broader system-level issues, including care coordination, workforce engagement, caregiver education, and digital infrastructure support.^59,60^

Timely postdischarge follow-up is recommended for nearly all hospitalized children, yet rural and socioeconomically disadvantaged populations remain at higher risk for postdischarge complications and have lower rates of timely postdischarge follow-up.^61,62,63^ Virtual care was expected to mitigate access to care barriers for these groups.^5,64^ However, we found that the implementation of a virtual care remuneration policy was not associated with a change in postdischarge follow-up rates for rural and more materially deprived populations. In addition, the increase in virtual care uptake was greater among urban and higher-resourced populations, a recognized pattern across various North American health care settings.^25,26,29,65,66^

Before the virtual care remuneration policy implementation, virtual care comprised 0.1% of postdischarge follow-up visits among the highest socioeconomic status quintile and 0.2% among the lowest quintile. After its implementation, virtual follow-up accounted for a higher proportion of follow-up visits in the highest socioeconomic status quintile compared with the lowest (11.8% vs 9.5%). Disparities in digital access, literacy, and infrastructure are likely associated with this inequitable uptake.^27,67,68,69^ Without targeted investment in digital infrastructure and inclusive care models, virtual care expansion may exacerbate, rather than reduce, disparities during the transition from hospital to home.^27,70^

In this study, the proportion of follow-up visits conducted virtually was lowest among medical admissions compared with surgical admissions in the current virtual care era. One possible explanation is the relative paucity of safety data for this patient group. Evidence from adult populations indicates comparable adverse event rates for virtual and in-person follow-up after surgical hospitalizations, whereas safety of virtual care in medical conditions is mixed.^5,71,72,73,74^ Rigorous, condition-specific studies in pediatric cohorts are required to clarify the safety profile of virtual postdischarge care to guide its appropriate use.

### Limitations

Our study has several limitations. Although we accounted for seasonality and autocorrelation, unmeasured system-level changes may have been associated with follow-up rates independent of the virtual care remuneration policy. However, by comparing the current and pre–virtual care eras, several system-level changes that occurred early in the COVID-19 pandemic had resolved, reducing the risk of confounding. Furthermore, no large-scale postdischarge follow-up interventions occurred in Ontario during the study period. We could not capture follow-up provided by nonphysician clinicians, such as nurse practitioners. However, in Ontario, physicians provide most of the pediatric postdischarge follow-ups. In addition, follow-up was not restricted to clinician types that aligned with hospitalization type; we believe most follow-ups within 7 days were associated with some aspect of the index hospitalization, as follow-ups by unaligned clinician types were rare. We also used billing and location codes as a proxy for telephone or video virtual care use; while billing and location codes are reliable markers of visit modality, they do not capture other forms of virtual care, such as email or written platforms, as these are not included in the virtual care remuneration policy. Decisions regarding the need for and modality of follow-up may be associated with clinical factors and caregiver concerns, which are not captured in administrative data. Similarly, other demographic factors potentially associated with differential access to care, such as race and ethnicity, were not available in the administrative data. Findings from a single-payer system in Ontario may not be generalizable to other jurisdictions. In addition, we did not evaluate for missed follow-up visits or for the appropriateness of follow-up visits, as they were not the focus of the virtual care policy.

## Conclusions

In this repeated cross-sectional study, the implementation of a virtual care remuneration policy in Ontario was not associated with a clinically meaningful increase in timely follow-up after pediatric hospitalization, nor was it associated with narrowing of geographic and socioeconomic disparities. Although virtual care use increased across all groups, the smallest gains were observed among children in rural and materially deprived areas—a population for whom virtual care was expected to make meaningful improvements to access to care. These findings suggest that virtual care billing policies alone may be insufficient to enhance follow-up in transitional care and may inadvertently reinforce existing inequities.

## Notes

Supplement 1. **eTable 1.** Proportion of Hospitalized Patients That Received a Post-Discharge Follow Up Visit Across Virtual Care Eras **eTable 2.** Number of Follow Up Visits Within 7-Days of Hospital Discharge, by Provider Type and Across Virtual Care Eras **eTable 3.** Proportion of Virtual 7-Day Post-Discharge Follow Up Visits Across Virtual Care Eras **eTable 4.** Interrupted Time Series Analysis Slope and Level Change in 7-Day Post-Discharge Follow Up Rates Following Onset of the System-Level Virtual Care Remuneration Policy **eTable 5.** Absolute Difference Between the Observed and Predicted 7-Day Post-Discharge Follow Up Visits in the Current Virtual Care Era **eTable 6.** Interrupted time Series Analysis of Slope and Level Change in Monthly Age- and Sex-Standardized 7-Day Post-Discharge Follow Up Rates Following Onset of Current Virtual Care Billing Codes **eTable 7.** Interrupted Time Series Analysis of Slope and Level Change of 7-Day Post-Discharge Follow Up Rates Following Onset of Current Virtual Care Billing Codes, When Excluding Patients Readmitted Within 7-Days of Discharge

Supplement 2.
Data Sharing Statement
