# Supplementary material for: Virtual Care Remuneration Policy and Postdischarge Follow-Up Trends
Source: JAMA Netw Open. 2026 Jun 24;9(6):e2620021. doi: 10.1001/jamanetworkopen.2026.20021 (PMC13294786; doi:10.1001/jamanetworkopen.2026.20021)
Supplement: Supplement 1. — eTable 1. Proportion of Hospitalized Patients That Received a Post-Discharge Follow Up Visit Across Virtual Care Eras eTable 2. Number of Follow Up Visits Within 7-Days of Hospital Discharge, by Provider Type and Across Virtual Care Eras eTable 3. Proportion of Virtual 7-Day Post-Discharge Follow Up Visits Across Virtual Care Eras eTable 4. Interrupted Time Series Analysis Slope and Level Change in 7-Day Post-Discharge Follow Up Rates Following Onset of the System-Level Virtual Care Remuneration Policy eTable 5. Absolute Difference Between the Observed and Predicted 7-Day Post-Discharge Follow Up Visits in the Current Virtual Care Era eTable 6. Interrupted time Series Analysis of Slope and Level Change in Monthly Age- and Sex-Standardized 7-Day Post-Discharge Follow Up Rates Following Onset of Current Virtual Care Billing Codes eTable 7. Interrupted Time Series Analysis of Slope and Level Change of 7-Day Post-Discharge Follow Up Rates Following Onset of Current Virtual Care Billing Codes, When Excluding Patients Readmitted Within 7-Days of Discharge [file jamanetwopen-e2620021-s001.pdf]

Supplementary Online Content

D'Arienzo D, Mahant S, Austin PC, Yoshida-Montezuma Y, Guttmann A. Virtual care remuneration policy and postdischarge follow-up trends. *JAMA Netw Open.* 2026;9(6):e2620021. doi:10.1001/jamanetworkopen.2026.20021

eTable 1. Proportion of Hospitalized Patients That Received a Post-Discharge Follow Up Visit Across Virtual Care Eras
eTable 2. Number of Follow Up Visits Within 7-Days of Hospital Discharge, by Provider Type and Across Virtual Care Eras
eTable 3. Proportion of Virtual 7-Day Post-Discharge Follow Up Visits Across Virtual Care Eras
eTable 4. Interrupted Time Series Analysis Slope and Level Change in 7-Day Post-Discharge Follow Up Rates Following Onset of the System-Level Virtual Care Remuneration Policy
eTable 5. Absolute Difference Between the Observed and Predicted 7-Day Post-Discharge Follow Up Visits in the Current Virtual Care Era
eTable 6. Interrupted time Series Analysis of Slope and Level Change in Monthly Age- and Sex-Standardized 7-Day Post-Discharge Follow Up Rates Following Onset of Current Virtual Care Billing Codes
eTable 7. Interrupted Time Series Analysis of Slope and Level Change of 7-Day Post-Discharge Follow Up Rates Following Onset of Current Virtual Care Billing Codes, When Excluding Patients Readmitted Within 7-Days of Discharge

This supplementary material has been provided by the authors to give readers additional information about their work.

© 2026 D'Arienzo D et al. *JAMA Network Open.*

eTable 1. Proportion of Hospitalized Patients That Received a Post-Discharge Follow Up Visit Across Virtual Care Eras

	**Follow Up within 7-days of Discharge**			**Follow Up within 14-days of Discharge**			**Follow Up within 30-days of Discharge**		
	Pre-VC care era n (%)	Temp. VC era n (%)	Current VC era n (%)	Pre-VC care era n (%)	Temp. VC era n (%)	Current VC era n (%)	Pre-VC care era n (%)	Temp. VC era n (%)	Current VC era n (%)
Annualized Discharges	50,429 (100)	40,883 (100)	50,514 (100)	50,429 (100)	40,883 (100)	50,514 (100)	50,429 (100)	40,883 (100)	50,514 (100)
Overall	21,185 (42.0)	17,121 (41.9)	20,645 (40.9)	30,008 (59.5)	23,915 (58.5)	28,945 (56.7)	37,965 (75.3)	30,503 (74.6)	36,780 (72.8)
**Sex**									
Female	9.351 (41.6)	7,811 (42.0)	9,244 (40.7)	13,260 (58.9)	10,850 (58.3)	12,854 (56.6)	16,805 (74.7)	13,842 (74.3)	16,449 (72.4)
Male	11,834 (42.4)	9,312 (41.8)	11,401 (41.0)	16,748 (60.0)	13,066 (58.7)	15,791 (56.8)	21,160 (75.8)	16,661 (74.8)	20,331 (73.2)
**Hospitalization Type**									
Medical	17,094 (47.4)	13,703 (46.3)	16,900 (44.2)	22,660 (62.8)	18,219 (61.5)	22,529 (59.0)	27,487 (76.2)	22,373 (75.5)	27,982 (73.2)
Surgical	4,061 (28.5)	3,536 (30.3)	3,751 (30.4)	7,348 (51.3)	5,909 (50.6)	6,128 (49.7)	10,478 (73.1)	8,433 (72.2)	8,835 (71.5)
**Rurality**									
Rural	1,074 (31.3)	856 (31.9)	960 (29.8)	1,689 (49.1)	1,303 (48.6)	1,484 (46.0)	2,244 (65.3)	1,786 (66.6)	2,018 (62.6)
Urban	20,110 (42.8)	16,265 (42.6)	19,685 (41.7)	28,320 (60.3)	22,612 (59.2)	27,161 (57.4)	35,721 (76.0)	28,717 (75.2)	34,762 (73.5)
**Material Resources Quintile***									
1 (least deprived)	3,957 (42.5)	3,132 (43.0)	3,622 (40.7)	5,627 (60.4)	4,386 (60.3)	5,078 (57.1)	7,141 (76.7)	5,542 (76.1)	6,584 (74.0)
2	4,135 (42.4)	3,730 (42.7)	4,529 (42.2)	5,885 (60.3)	5,223 (59.7)	6,267 (58.5)	7,432 (76.2)	6,645 (76.0)	8,018 (74.8)
3	3,915 (41.9)	3,390 (42.5)	4,210 (41.8)	5,587 (59.8)	4,725 (59.2)	5,816 (57.6)	7,064 (75.6)	6,051 (75.8)	7,440 (73.7)
4	4,000 (42.5)	3.030 (41.7)	3,684 (41.0)	5,611 (59.7)	4,194 (57.8)	5,085 (56.6)	7,078 (75.3)	5,388 (74.2)	6,543 (72.8)
5 (most deprived)	5,177 (41.0)	4,839 (39.9)	4,601 (39.0)	7,298 (57.9)	5,387 (56.0)	6,399 (54,2)	9,249 (73.4)	6,876 (71.5)	8,196 (69.4)

n presented is annualized.
VC: Virtual Care; Temp.: Temporary; Pre-virtual care era (March 1, 2011 to February 29, 2020); Temporary virtual care era (June 1, 2020 to November 30, 2022); Current virtual care era

© 2026 D'Arienzo D et al. *JAMA Network Open.*

(December 1, 2022 to June 30, 2024). The current virtual care era represents the onset of the system-level virtual care remuneration policy.
*Material Resource, a marker of neighbourhood socioeconomic status, combines income, unemployment rates, household condition, education-level, and single-parent family rates.

© 2026 D'Arienzo D et al. *JAMA Network Open.*

eTable 2. Number of Follow Up Visits Within 7-Days of Hospital Discharge, by Provider Type and Across Virtual Care Eras

	**Pre-virtual care era (March 1, 2011 to February 29, 2020)**		**Temporary virtual care era (June 1, 2020 to November 30, 2022)**		**Current virtual care era (December 1, 2022 to June 30, 2024)**	
	**Medical**	**Surgical**	**Medical**	**Surgical**	**Medical**	**Surgical**
Median days to follow up (IQR)	5 (2-11)	10 (5-16)	5 (2-12)	9 (4-16)	5 (2-12)	9 (4-17)
**Follow up provider, n (%)**						
General practitioner	48,276 (31.7)	10,028 (27.5)	9,101 (27.0)	2,188 (26.0)	6,898 (26.2)	1,276 (21.8)
Pediatrician	82,444 (54.2)	7,444 (21.5)	18,993 (56.4)	1,935 (23.0)	15,538 (58.9)	1,150 (19.6)
Surgeon	11,404 (7.51)	17,496 (47.1)	2,345 (7.0)	3,823 (45.2)	1,816 (7.0%)	2,567 (43.8)
Other	9,973 (6.6)	1,432 (3.9)	3,248 (9.6)	487 (5.8)	2,126 (7.9)	863 (14.7)

The first post-discharge follow up included per observation.
Surgical providers include general surgery, otolaryngology, orthopedic surgery, cardiothoracic surgery, vascular surgery, plastic surgery, neurosurgery, urology, dental/oral surgery, obstetrics/gynecology, and anesthesia
Other providers included psychiatry, endocrinology, nephrology, neurology, psychiatry, genetics, cardiology, hematology, nuclear medicine, ophthalmology, rheumatology, medical oncology, palliative medicine, infectious disease, respirology, physical medicine, dermatology, diagnostic radiology, radiation oncology, internal medicine.

© 2026 D'Arienzo D et al. *JAMA Network Open.*

eTable 3. Proportion of Virtual 7-Day Post-Discharge Follow Up Visits Across Virtual Care Eras

	**Pre-Virtual Care Era (Mar 1, 2011- Feb 29, 2020)** n (%)	**Temporary Virtual Care Codes Era (June 1, 2020 – Nov 30, 2022)** n (%)	**Current Virtual Care Era (Dec 1, 2022 - June 30, 2024)** n (%)
Annualized All-Modality Follow Up Visits	21,185	17,121	20,645
Annualized Virtual Follow Up	34 (0.16)	5,006 (29.7)	2,247 (11.0)
**Hospitalization Type**			
Medical	28 (0.17)	3,975 (29.0)	1,732 (10.3)
Surgical	6 (0.15)	1,070 (30.6)	516 (13.9)
**Rurality**			
Rural	6 (0.60)	193 (22.5)	103 (10.7)
Urban	28.1 (0.14)	4,814 (29.6)	2,144 (10.9)
**Material Resource Quintile***			
1 (least deprived)	4 (0.10)	9.47 (30.3)	428 (11.8)
2	7 (0.16)	1,142 (30.6)	531 (11.7)
3	6 (0.16)	1,020 (30.8)	466 (11.1)
4	8 (0.19)	868 (28.6)	384 (10.4)
5 (most deprived)	10 (0.19)	1,030 (26.8)	438 (9.5)

n presented is annualized
*Material Resource a marker of neighbourhood socioeconomic status, which combines income, unemployment rates, household condition, education-level, and single-parent family rates

© 2026 D'Arienzo D et al. *JAMA Network Open.*

eTable 4. Interrupted Time Series Analysis Slope and Level Change in 7-Day Post-Discharge Follow Up Rates Following Onset of the System-Level Virtual Care Remuneration Policy

	Pre-Virtual Care Era Slope (95% CI)		Current Virtual Care Era Slope (95% CI)		Slope Difference		Level Change	
					Parameter Estimate	95% CI	Parameter Estimate	95% CI
**Entire Cohort**	0.28	(0.02, 0.43)	0.58	(-0.13, 1.29)	0.36	(-0.15, 0.87)	-2.70	(-7.09, 1.69)
**Hospitalization Type**								
Medical	0.35	(-0.07, 0.76)	1.24	(0.55, 1.93)	0.89*	(0.18,1.60)	-6.51*	(-0.003, -13.02)
Surgical	-0.24	(-0.38, -0.11)	0.41	(0.08, 0.74)	0.65*	(0.22, 1.08)	7.86*	(3.94, 11.78)
**Rurality**								
Rural	0.09	(-0.28, 0.44)	0.36	(-1.05, 1.77)	0.27	(-0.85, 1.39)	-0.66	(-1.70, 0.38)
Urban	0.22	(0.01, 0.43)	0.58	(0.09, 1.07)	0.36	(-0.15, 0.87)	-2.80	(-7.27, 1.67)
**Material Resource Score**+								
Highest Material Resource (Most Deprived)	0.10	(-0.07, 0.28)	-0.02	(-0.15, 0.11)	-0.12	(-1.14, 0.90)	1.86	(-9.66, 13.38)
Lowest Material Resource (Least Deprived)	0.33	(0.06, 0.59)	0.72	(0.13, 1.31)	0.39	(-0.28, 1.05)	-3.40	(-9.03, 2.22)

Slopes are presented as monthly rates per 1,000 hospital discharges. Level change is rate per 1,000 hospital discharges. ARIMA models adjusted for autocorrelation and seasonality. Pre-virtual care era from March 1, 2011-Feb 29, 2020 reflects a period of minimal virtual care use and highly restrictive virtual care billing codes. Current virtual care era from Dec 1, 2022-June 20, 2024 reflects the onset of the system-level virtual care remuneration policy. CI: Confidence Interval

*Statistically significant at p-value <0.05

+Material Resource, a marker of neighbourhood socioeconomic status, combines income, unemployment rates, household condition, education-level, and single-parent family rates

© 2026 D'Arienzo D et al. *JAMA Network Open.*

eTable 5. Absolute Difference Between the Observed and Predicted 7-Day Post-Discharge Follow Up Visits in the Current Virtual Care Era

	Predicted Mean Monthly Follow Up Rate	95% Confidence Intervals	Observed Mean Monthly Follow Up Rate	Difference in Observed-Predicted Follow Ups per 1,000 Hospital Discharged
**Entire Cohort**	41.0%	(0.38-0.44)	40.9%	-1
**Hospitalization Type**				
Medical	44.8%	(0.41-0.49)	44.4%	-4
Surgical	26.4%	(0.23-0.29)	30.4%	+43
**Rurality**				
Rural	30.1%	(0.22-0.38)	29.8%	-3
Urban	41.9%	(0.38-0.45)	41.7%	-2
**Material Resource Score***				
Highest Material Resource (Most Deprived)	40.0%	(0.36-0.44)	40.0%	-1
Lowest Material Resource (Least Deprived)	41.7%	(0.39-0.46)	41.7%	0

Overall and for all subgroups, the observed follow up rates fell within the 95% confidence intervals of the predicted rates, indicating no statistically significant differences.

*Material Resource, a marker of neighbourhood socioeconomic status, combines income, unemployment rates, household condition, education-level, and single-parent family rates.

© 2026 D'Arienzo D et al. *JAMA Network Open.*

eTable 6. Interrupted time Series Analysis of Slope and Level Change in Monthly Age- and Sex-Standardized 7-Day Post-Discharge Follow Up Rates Following Onset of Current Virtual Care Billing Codes

	Pre-Virtual Care Era Slope (95% CI)		Current Virtual Care Era Slope (95% CI)		Slope Difference		Level Change	
					Parameter Estimate	95% Confidence Interval	Parameter Estimate	95% Confidence Interval
**Entire Cohort**	0.19	(-0.06, 0.43)	0.48	(-0.25, 1.21)	0.29	(-0.22, 0.80)	-1.69	(-6.19, 2.81)
**Hospitalization Type**								
Medical	0.30	(-0.13, 0.73)	0.57	(-0.23, 1.37)	0.27	(-0.63, 1.18)	-5.66	(-15.64 , 6.31)
Surgical	-0.26	(-0.39, -0.12)	0.42	(-0.09, 0.93)	0.68*	(0.15, 1.20)	6.28*	(1.12, 11.43)
**Rurality**								
Rural	0.01	(-0.36, 0.38)	0.22	(-0.01, 0.44)	0.21	(-1.01, 1.44)	0.63	(-10.38, 11.65)
Urban	0.18	(-0.05, 0. 42)	0.49	(-0.06 , 1.04)	0.31	(-0.22, 0.85)	-1.96	(-6.64, 2.72)
**Material Resource Score+**								
Highest Material Resource (Most Deprived)	0.04	(-0.08, 0.17)	0.12	(-0.33, 0.57)	0.16	(-0.38,0.70)	-0.45	(-5.25, 4.36)
Lowest Material Resource (Least Deprived)	0.31	(-0.37-, 0.66)	0.39	(-0.02, 0.78)	0.08	(-0.85, 1.01)	0.21	(10.12, 10.54)

Slopes are presented as monthly rates per 1,000 hospital discharges. Level change is rate per 1,000 hospital discharges. ARIMA models adjusted for autocorrelation and seasonality.
Pre-virtual care era from March 1, 2011- Feb 29, 2020; Current virtual care era from Dec 1, 2022- June 20, 2024
*Statistically significant at p-value <0.05
+Material Resource a marker of neighbourhood socioeconomic status, which combines income, unemployment rates, household condition, education-level, and single-parent family rate

© 2026 D'Arienzo D et al. *JAMA Network Open.*

eTable 7. Interrupted Time Series Analysis of Slope and Level Change of 7-Day Post-Discharge Follow Up Rates Following Onset of Current Virtual Care Billing Codes, When Excluding Patients Readmitted Within 7-Days of Discharge

	Pre-Virtual Care Era Slope (95% CI)		Current Virtual Care Era Slope (95% CI)		Slope Difference		Level Change	
					Parameter Estimate	95% Confidence Interval	Parameter Estimate	95% Confidence Interval
**Entire Cohort**	0.21	(0.01, 0.42)	0.53	(-0.02, 1.08)	0.32	(-0.19, 0.82)	-2.39	(-6.84 , 2.06)
**Hospitalization Type**								
Medical	0.34	(-0.08, 0.76)	0.70	(-0.32, 1.72)	0.36	(-0.50, 1.22)	-1.54	(-10.88, 7.79)
Surgical	-0.26	(-0.38, -0.13)	0.59	(-0.81, 0.90)	0.85*	(0.50, 1.20)	7.14*	(3.16, 11.12)
**Rurality**								
Rural	0.08	(-0.29, 0.45)	0.15	(-0.24, 0.54)	0.07	(-1.05, 1.19)	1.03	(-8.78, 10.85)
Urban	0.21	(-0.08, 0.76)	0.55	(-0.43, 1.53)	0.34	(-0.19, 0.86)	-2.63	(-7.23, 1.96)
**Material Resource Score**[+]								
Highest Material Resource (Most Deprived)	0.09	(-0.08, 0.27)	0.56	(-0.50, 1.62)	0.47	(-0.41, 1.35)	-3.82	(-12.41, 4.78)
Lowest Material Resource (Least Deprived)	0.31	(0.05, 0.57)	0.57	(-0.04, 1.18)	0.26	(-0.33, 0.86)	-1.86	(-7.25, 3.52)

Slopes are presented as monthly rates per 1,000 hospital discharges. Level change is rate per 1,000 hospital discharges. ARIMA models adjusted for autocorrelation and seasonality.
Pre-virtual care era from March 1, 2011- Feb 29, 2020; Current virtual care era from Dec 1, 2022- June 20, 2024
*Statistically significant at p-value <0.05
[+]Material Resource a marker of neighbourhood socioeconomic status, which combines income, unemployment rates, household condition, education-level, and single-parent family rate

© 2026 D'Arienzo D et al. *JAMA Network Open.*
